# Relationship between Homesickness and Test Anxiety in Non-Native Students of Shiraz University of Medical Sciences International Branch in the Clinical and Physiopathology Course In 2013

**DOI:** 10.5539/gjhs.v8n7p293

**Published:** 2015-12-16

**Authors:** Saman Azizi

**Affiliations:** 1 School of Medicine, Shiraz University of Medical Sciences, Shiraz, Iran

**Keywords:** homesickness, exam anxiety, non-native students

## Abstract

**Introduction:**

Anxiety is an emotional and physiological response to the internal felling of overall danger that is easily resolved. The aim of this study has been to determine the relationship between exam anxiety and the feeling of homesickness among non-native students.

**Methodology:**

The present study is cross-sectional and the subjects in this study are 80 non-native male and female PhD candidates in clinical and physiopathology majors in 2013 academic year that have been evaluated with the help of Persian homesickness questionnaire and Sarason’s test anxiety questionnaire and the data was analyzed using Pearson’s correlation coefficient.

**Results:**

With regard to the Pearson’s correlation coefficient there is a significant and reverse relationship between the desire to return to home and exam anxiety (r=0.0344, p=0.004) and there is a significant and reverse relationship between the Compatibility and exam anxiety (r=0.428, p<0.0001) and there is a significant and direct relationship between the feeling of alone and exam anxiety (r=0.888, p<0.0001).

**Discussion & Conclusion:**

There is a significant relationship between the feeling of homesickness and exam anxiety and the mental health of non-native students will be deteriorated by the feeling of homesickness and anxiety.

## 1. Introduction

Anxiety is a feeling of extended, unpleasant and vaguely fear (panic) and anxiety, of unknown origin that a person feels, such as uncertainty, helplessness and physiological provocation. The recurrence of situations that are stressful or over which individuals have already been hurt, causes anxiety in people. The prevalence of anxiety states in the normal population in England and America is estimated to be 2 to 4.7%. However, an assessment of the figure mentioned is about 21% in the Framingham area. Anxiety disorders were noticed in 14% of patients with heart disease and 22% of patients who have been operated. Studies show that more than a third of adults are with neurological disorders, especially anxiety, and anxiety in men and economic affluent class is less and it is more in women and youth, and low-income people and the elderly. Test anxiety is a situational anxiety is closely related to the performance and achievement of millions of students in educational centers. When a person with respect to efficiency, ability and aptitude is tested in test conditions or in situations where he is evaluated, he is filled with worry, anxiety and hesitation that can be discussed about as test anxiety. A homesickness feeling is a kind of feeling of loneliness, isolation or confusion that occur because of the failure and separation from the environment, people, traditions and rituals that they used to. This condition can occur for all people in all age groups but generally speaking happens mostly in children and adolescents when their previous environment (such as former school or former neighborhood) is left. This condition is seen also in the early months of migration, especially among women more than men. Especially those children in puberty period who are in amid of emotional and romantic experiences and refused immigration or their ideas have not been considered in this case they are also more likely to get involved with this phenomenon. Freshmen College or university students especially when they are educating in another city or another country, usually pass this experience. Among immigrants, the problem may mix with being concerned with the consequences of immigration and how to overcome the problems faced but these two things are distinct. Those who are nostalgic are usually sad and depressed. The incidence of these symptoms in children is often more… The symptoms include switching away from the others, refusal of participation in activities or actions performed to draw attention. Other symptoms include crying, insomnia and physical discomfort such as stomach pain, sore throat, headache, nausea and cold symptoms that can be noted. In order not to go to school, they may even go feigning their disease. People who are homesick they rarely need to see a doctor. Negative feelings of nostalgia, gradually is lost the through passing time and the person becomes accustomed to the new environment and feel at the home. For example, children who feel homesick in the night camp day or youth who are nostalgic after starting a course in another city, probably, after getting to know new friends did not notice at all the loss of their emotional states. In order to determine the relationship between test anxiety, homesickness and non-aboriginal medical students of Shiraz University of Medical Sciences the at International physiopathology and clinical units in 92 year, in this study, we are to find that if the residence is away from your native home, does it have any impact on homesickness of test anxiety or not the results of which need to be mindful enough to be used in strategies to reduce test anxiety and increase students’ adaptation to the residential environment and improve educational condition of the students.

## 2. Method

### 2.1 Research Methodology

This study was a cross-sectional study and the data were gathered through questionnaire.

### 2.2 Sample Population

The population of the study is all non-local students of the international school and clinical physiopathology units of Shiraz University of Medical Sciences and the sample under study is physiopathology and clinical medical students. In this research project, the student presenter has presence in different places such as training classrooms, the educational hospital of Shiraz University of Medical Sciences International unit. While explaining the importance of the project and its practical implementation and gathering verbal consent, he presents the questionnaires provided for them and after completing the questionnaire will be delivered from the students the next day.

### 2.3 The Research Sample

The sample included 90 medical students in physical education and clinical pathology level.

### 2.4 Research Instruments

1). The demographic questionnaire, in which features such as age, sex, marital status, year of entering the university and place of residence is asked and an example is presented in Appendix 1.
2). The students’ homesickness questionnaire, a questionnaire with 36 items which are a subset of five main factors. These 5 factors include nostalgia for the family, the desire to return home, adaptability, loneliness, longing for people met and the known environment. Questions are prepared based on a Likert scale of 5 points. In a way that the subject by selecting one of the options Never, a little, medium, high and very high, respectively, 0, 1, 2, 3, 4 and 5 will be scored. To obtain scores for each subscale, material inserted in front of each of them will be added together and in order to obtain a total score of homesickness, all subscale scores should be collected together; meanwhile, the questionnaire contains three additional component that the first two measures the abundance of homesickness experience on the current situation and in the past and the last component part measures the intensity of homesickness.
For validation of the questionnaire, the questionnaire technique taken from Fen Fylt is used that has been confirmed in the study conducted by J. Aegean and colleagues in 2008 which is presented in Appenix 2.
3). The questionnaire contains 25 questions with 4 options (Never=0, rarely=1, sometimes=2, often=3), the minimum score is zero and a maximum will be 75, the higher the score indicates greater anxiety. Thus, scoring less than 12 is non-indicative of anxiety, the scores between 13 and 37 shows low anxiety, between 38 to 63 average anxiety and scores above 63 are considered as high anxiety.

Validity and reliability of the questionnaire used in this study are previously determined that reliability re-tested equals 88%, the internal consistency 95% and the validity criteria equals 72% each of which is obtained and listed in Appendix 3.

### 2.5 The Data Collection Manner

Questionnaire is presented to 90 students and the necessity of research is explained to them and they are asked to carefully respond to questions.

### 2.6 Data Analysis

In order to analyze the data, descriptive statistics’ indices were used including, percentage frequency, mean, and tables. To determine the relationship between independent and dependent (anxiety) variables, Pearson correlation was examined. All data were analyzed through SPSS software (ver.15) in this study.

## 3. Results

### 3.1 The Results of the Statistical Analysis

90 questionnaires were distributed among medical students entering university in 2007, 2008, 2009, 2010, and 2011. Among which 80 questionnaires were authentic. The average age of the subjects was 24 and it is mentioned in Table1. The subjects were divided into clinical students and physiopathology students and were all living in dormitories or rented houses. The data obtained from the questionnaires were statistically analyzed. The first step to implement factor analysis on some set of items, was to use Pearson’s test. 36 items of homesickness questionnaire were analyzed through exploratory factor analysis and principal components analysis. By examining the content of factors, h1 was attributed to the first factor “nostalgia for the family”. This factor contains 10 items that are related to the concept of nostalgia for home and family (Soleimani, 2005; Abolqasemi et al., 2004; Van vliet, 2001; Burt, 1993; Azais Granger, Debray, & Ducroxi, 1991; Sobhi Gharamaki, 2006; Turner, 1993; Betz, 1978). The second factor is the content of the thinking of the past, “the desire to return to the homeland” or is called h2. This factor includes 10 items (Abolghasemi, Mehrabizadeh, Kyamarsi, & Azar, 2006; Frydkht & Bahram, 2011). The third factor is related to the issue of compromising on the new situation and is called adaptation or h3. This factor includes 8 items (Nijhof & Engels, 2007; Abolghasemi et al., 2006; Benn Harvey, Gilbert, & Irons, 2005; Archer, Irland, Amus, Broad, & Currid, 1998; Carr, Koyama, & Thiagarajan, 2003; Covington & Omelich, 1987; Yeh & Inose, 2003; Van Tilburg, Vingerhots, & van Heck, 1996). Items related to the fourth factor or feelings of isolation are called “loneliness” or h4 and contains 4 items (Yeh & Inose, 2003; Sarason, 1988; Van Tilburg, Vingerhots, & van Heck, 1996; Rajapaksa & Dundes, 2014). Finally, Factor 5 being “nostalgia for familiar people and the environments” is called h5 and contains 4 factors (Benn, Harvey, Gilbert, & Irons, 2005; Ogden, 2004; Messina, 2007; Tabeh Bordbar, Rasoulzadeh Tabatabai et al., 2008).

**Table 1:** The Determination of the age range of students participating in the project

Gender	MEAN	N	Std. Deviation
Male	**24.7333**	**30**	**3.13966**
Female	**24.12**	**50**	**2.31799**

**Figure 1:**
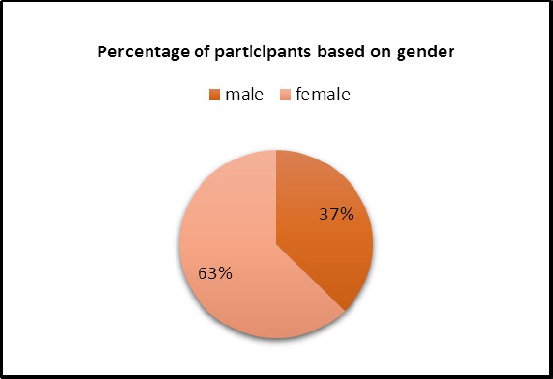
The percentage of students participating in the project on the basis of gender

Students participating in the project, included 37.5% male and 62.5% female, which are demonstrated in Figure 1.

**Figure 2:**
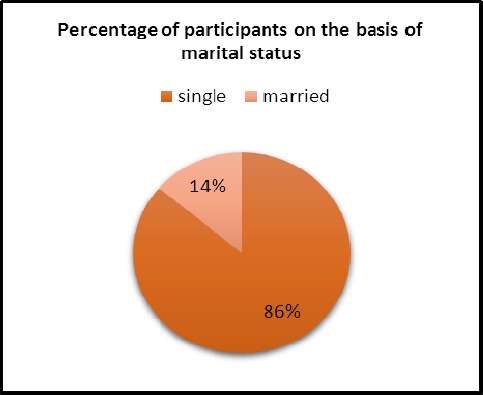
The percentage of students participating in the project based on marital status

86.3% were single and 13.8% were married. It is shown in Figure 2.

**Figure 3:**
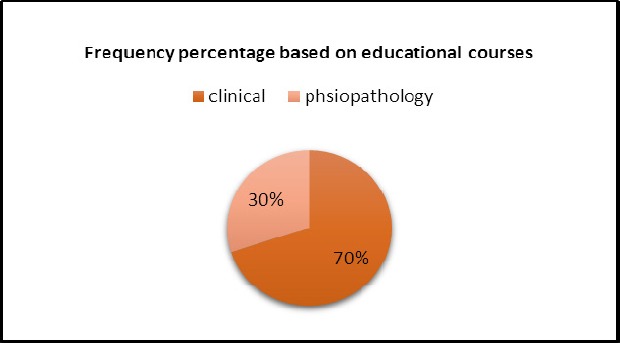
The Frequency percentage of students participating in the project based on educational courses

70% were studying clinical courses and 30% were studying physiopathology which is shown in Figure 3.

**Figure 4:**
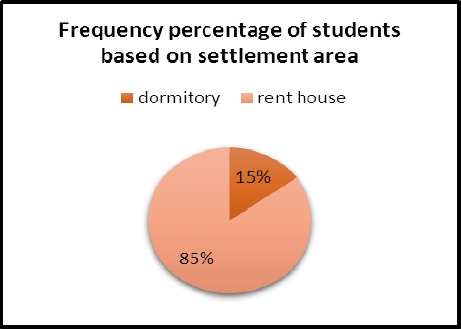
The Frequency percentage of students participating in the project based on settlement area

15% of students lived in dormitories and 85% of them lived in rented houses that is shown in pie chart Figure 4.

According to Pearson correlation there is a negative significant relationship between stress and the desire to return to the homeland, r=-0.334 and p=0.004. There is a negative significant relationship between anxiety and adaptation r=-0.428 ad p<0.0001. There is a significant relationship between loneliness and anxiety r=0.888 and p<0.0001. On the contrary, there exists a negative significant relationship between anxiety and the desire to return to homeland, although it was expected that the more the desire to return to homeland, the more the anxiety of the student. The justification is perhaps this fact that more anxious students try to adapt with the environment to overcome their stress and prefer to stay far from the family. To prove this assumption, another research could be conducted with a greater population and in other universities of the country. There is a negative significant relationship between anxiety and nostalgia for the family r=-0.295, p=0.049, the desire to return to the home (p=0.006, r=-0.403) and adaptation (p<0.001, r=-0.453) in females. However, there is a direct significant relationship between loneliness and anxiety (p<0.001, r=0.912). There is a direct significant relationship between anxiety and loneliness in males (p<0.001, r=0.840).

There is a negative significant relationship between anxiety and the desire to return to homeland (r=-0.319, p=0.011), and adaptation (r=-0.463, p<0.001) in singles. There is a direct significant relationship between anxiety and loneliness in singles (r=0.880, p<0.001). There is a direct significant relationship between anxiety and loneliness in married people (r=0. 947, p<0.001).

There is a negative significant relationship between anxiety and desire to return to homeland (p=0.003, r=-0.422), adaptation (p=0.002, r=-0.428) in clinical students. While, there is a significant relationship between anxiety and loneliness in the clinical students (r=0.886, p<0.001). There is a Negative significant relationship between anxiety and adaptation at physiopathology students (p=0.038, r=-0.444). While there is a direct significant relationship between anxiety and loneliness in the physiopathology students (p <0.001, r=0.891).

There is a significant negative relationship between anxiety and the desire to return to the homeland (r=-0.345, p=0.007), and adaptation (r=-0.411, p=0.001) in those students who rented a house. While there is a direct significant relationship between anxiety and loneliness in the same students (p <0.001, r=0.873). There is a significant relationship between loneliness and anxiety in students who lived in dormitories (p<0.001, r=0.952).

## 4. Discussion

Student living is a new situation that has its own tensions for newly arrived students. Students, especially girls, are faced with many cultural and educational challenges in dormitories. Accepting new roles, educational demands, and financial problems, loss of self-confidence, home sickness and defects in study skills, low courage during social interactions, high anxiety and tension are many examples of these problems. Homesickness is a special complicated cognitive, motivational and emotional situation that is associated with many mental preoccupations about the previous environment and the tendency to return to it and usually can be experienced with depressed mood and different symptoms of psychosomatics (Van Tilburg, Vingerhots & van Heck, 1996). Based on the report of the health association of students in America, the 2 first weeks of entering to the university is a very critical period of time for the process of compatibility in students. Students, who suffer from the feeling of homesickness, are more dissatisfied about studying in university and academic problems and the possibility of dropping out of school in them is three times more than the normal population (Yeh & Inose, 2003). Homesickness can lead to the shortage of reassuring relationships and feelings of loneliness and predisposing the risk of anxiety, depression and consumption of drug and alcohol and even suicide (Archer, Irland, Amus, Broad & Currid, 1998). Many researchers believe that most of the students, who suffer from homesickness, are captive in their weakness and do not act courageously in the field of social interaction, especially in making connection and receiving support from others (Azais, Granger, Debray & Ducroxi, 1991). This group will feel a lot of stress and anxiety in facing with stressors and will lose the ability to think and make compatibility in a constructive and useful way and as a result, they will act very passively in the new environment (Burt, 1993). In general, it can be said that the lack of self-esteem and low courage are characteristics of individuals with homesickness. This lack of self-confidence in those with homesickness can lead to the desire to avoid social contacts (Benn, Harvey, Gilbert & Irons, 2005). According to conducted researches, students with homesickness, besides having a high rate of depression and anxiety, have the tendency toward harsh isolation and to stay away from the community and they do not express their feelings (Ogden, 2004).

In recent years, many studies have been done to determine the relationship between homesickness and the amount of perceived support of the newly arrived students. Research results have shown that there is a direct and significant relationship between data obtained from the total score of homesickness and mental health and also, compatibility variables, the scale of the entire social protection, nostalgia for the family and feelings of loneliness can respectively predict the amount of mental health in newly arrived students. The results showed that the mental health of newly arrived students will increase with higher levels of perceived social support and will be reduced with experiencing more homesickness. Previous studies showed that there is a reverse and significant relationship between the total score of social support and the score of mental health and also, there is a direct and significant relationship between the total score of homesickness and mental health.

The results showed that the mental health of newly arrived students will increase with higher levels of perceived social support and will be reduced with experiencing more homesickness. Results of regression analysis, based on stepwise method also showed that compatibility variables, the scale of the entire social protection, nostalgia for the family and feelings of loneliness can respectively predict the amount of mental health in newly arrived students (Messina, 2007). In justifying these findings it can be said that social relations, approximately all the time, can be a shield against the pressing events that students experience and their absence is stressful and creates problems in compatibility. Social support can decrease the social isolation and provides a sense of self-esteem and feelings of worthiness in the person; other researchers have also reported that whatever the person obtain higher levels of social support from others, proportionally, he will benefit from higher levels of mental health. As it was stated, newly arrived students have more anxiety in comparing with older students and will face with more mental challenges. Chronic and severe homesickness can be associated with severe cognitive problems including memory mistakes and poor concentration and lead to academic problems of students (Van vliet, 2001). Investigating the phenomenon of nostalgia with therapeutic interventions in order to prevent the intensity and prevalence of it has always been considered by researchers. The results of existing researches have emphasized on interventions such as assertiveness training and having the courage, detente training and social skills training, controlled breathing, stop thinking, doing favorite activities and teaching the coping skills (Tabeh Bordbar, Rasoulzadeh Tabatabai & et al, 2008).

Many studies were performed in recent years to determine the effectiveness of cognitive-behavioral intervention of students in reducing the test anxiety. The multiplicity of writings and conducted studies in the last 50 years, in the field of the amount of prevalence and different treatments for test anxiety shows the importance and special attention of different countries towards this global phenomenon and conducting researches in the field of the impact of psychological treatments in reducing test anxiety. Test anxiety is a phenomenon that has a long and rich history in research. Initial researches related to test anxiety has begun since 1914 (Stopper & Pekron, 2004, quoted by Gharamleki). The phenomenon of test anxiety is among the most common educational issues. This phenomenon had a negative correlation with academic performance and is one of the main causes of academic failure. Test anxiety is an unpleasant experience that affects the cognitive, physiological and emotional fields. In general, it can be said that test anxiety plays a negative and deleterious role in the physical and mental health of students and may affect their academic performance. Many reasons and factors may cause test anxiety (Abolqasemi et al., 2004). Soleimani in 2005 carried out a research on the frequency of test anxiety in students and its relationship with academic performance and showed that the frequency of anxiety is reported between 10 and 30 percent and he indicated that there is a reverse relationship between the average score of test anxiety and academic performance (Soleimani, 2005).

Results of the Betz’s study (1987) found that math test anxiety is very common among students and this issue is especially true about girls who have more test anxiety than boys which this is still in line with the current study (Betz, 1978). Turner’s (1993) study showed that 41 percent of elementary students had test anxiety (Turner, 2001). Children who have test anxiety underestimated their cognitive and social abilities and have lower sense of self-worth. The results of McDonald’s revision (2001) shows that the prevalence rate of test anxiety is increasing day by day due to the growing use of tests for assessing the performance of individuals. Studies showed that the mean of test anxiety in girls is more than boys. About the prevalence of test anxiety in girls, both genetic factors and environmental factors can be noted. Research evidence indicates that the following factors play a major role in explaining and providing medical solutions for test anxiety. Individual and familial, scholastic and environmental factors of results of some studies show that individual factors have more influence among the top four factors. Among individual factors, many cognitive and personality characteristics, including the gender (being female), low self-esteem, low self-efficacy, the external control source, high public anxiety, distraction, personal irrational expectations and feelings of helplessness have been reported as the causes of test anxiety. Cognitive and personality factors can have an impact on stressful events, selection of coping patterns and behavioral and physiological reactions and in such circumstances; self-esteem will be damaged and especially can lead to anxiety in the person during the test (McDonald, 2001). In other words, the person has the background of anxiety (attribute or streaks), but it will be manifested during the exam and further evaluation. This issue is mentioned as one of the reasons of the frequency of test anxiety prevalence (especially girls). This study showed that gender and behavioral interventions were effective in creating test anxiety in students. In the mid-1960s, the use of relaxation and regular desensitization for the treatment of test anxiety has been emphasized by researchers. In the past two decades, cognitive techniques have been more emphasized. More recently, cognitive-behavioral interventions have been very effective in reducing test anxiety (Abolghasemi et al., 2006). A study was carried out by Faridokht Yazdani and colleagues in the academic year of 2011 to examine test anxiety and its relationship with the academic performance of midwifery students. They stated that anxiety is one of the emotional feelings of humans, but when this feeling is intensified; it may lead to undesirable results. Taking exams is one of the most threatening events that nowadays can lead to anxiety among students. This study investigates the relationship between test anxiety and academic performance of midwifery students of Islamic Azad University of Najafabad unit. Their study such as the present study was a cross sectional and in descriptive-analytical terms that was conducted on 114 persons of midwifery students in 2011. The data gathering tool included two background questionnaires with 14-items and the Sarason anxiety test with 37-item that in the present study, we used the same questionnaire.

They analyzed the data with Pearson-Spearman correlation coefficient and concluded that due to the high test anxiety in midwifery students and its inverse and significant relationship with academic performance, helping students for effective management of anxiety is a challenging task that requires a team effort. In their study, there was a significant relationship between anxiety and academic performance but was no significant relationship between anxiety and marital status, although the level of anxiety in single individuals was more than married students in their study which is exactly similar with the current study. In their research, incoming students (starting in September and February) don’t have the same level of anxiety and anxiety is not the same in different terms and anxiety isn’t related to the levels of school, but in some terms and different entrances, anxiety is higher than the rest which is consistent with the present study that is higher in clinical section than physiopathology section (Frydkht & Bahram, 2011).

## 5. Conclusion

There is a significant relationship between the components of homesickness and test anxiety and the mental health of non-native students will decrease by creating a feeling of homesickness and anxiety. Therefore, surveying the mental health of non-local students and investigating strategies to reduce their anxiety is an important component.

## References

[ref1] Abolghasemi A, Mehrabizadeh H, Kyamarsi M, Azar D. F (2006). An evaluation of the effectiveness of two logical-emotional methodology in reducing test anxiety and improvement academic performance. Andishehaye Novin Tarbiati journal.

[ref2] Abolqasemi A (2004). Effectiveness of two methods for reducing anxiety. Journal of Psychology.

[ref3] Archer J, Irland J, Amus S. L, Broad H, Currid L (1998). Duration of Homesickness scale. British J Psychol.

[ref4] Azais F, Granger B, Debray Q, Ducroxi C. X (1991). Cognitive and emotional approach to assertiveness. L'encephale.

[ref5] Benn L, Harvey J. E, Gilbert P, Irons C (2005). Social rank, Interpersonal trust and recall of parental rearing in relation to homesickness.

[ref6] Benn L, Harvey J. E, Gilbert P, Irons C (2005). Social rank, interpersonal trust and recall of parental rearing in relation to homesickness. Personality and individual differences.

[ref7] Betz N (1978). Prevention, distribution and correlates of math anxiety in college students. Journal of counseling psychology.

[ref8] Burt C. D. B (1993). Concentration and academic ability following transition to university: An investigation elderly men. Psychological Med.

[ref9] Carr J. L, Koyama M, Thiagarajan M (2003). A women's support group for Asian international students. J Am College.

[ref10] Covington M, Omelich C (1987). I knew it could before the exam: A test of anxiety-blockage hypothesis. Journal of educational psychology.

[ref11] Frydkht Y, Bahram S (2011). Evaluation of test anxiety and its relationship with students'academic performance. Journal of Health System Research.

[ref12] Mcdonald A (2001). The prevalence and effects of test anxiety in schoolchildren. Educational psychology.

[ref13] Messina J (2007). Helping students cope with Homesickness. University Business.

[ref14] Nijhof K. S, Engels R. C. M. E arenting styles, coping strategies, and the expression of homesickness. Journal of Adolescence.

[ref15] Rajapaksa S, Dundes L (2014). It's a long way home:international student adjustment to living in the United States. College Student Retention.

[ref16] Sarason I (1988). Anxiety, self-preoccupation and attention. Routledge.

[ref17] Soleimani M. J (2005). The frequency of test anxiety and its relationship with academic performance of male and female school students in Zahedan, public doctoral thesis. University of Medical Sciences.

[ref18] Tabeh Bordbar F. K, Rasoulzadeh Tabatabai (2008). Effects of courage and inoculation on homesickness in students. Journal of Behavioral Sciences.

[ref19] Turner B (1993). Test anxiety in African American schools psychology Quarterly.

[ref20] Van Tilburg M, Vingerhots Ad, van Heck G. L (1996). Homesickness: A review of the literature. Psychological medicine.

[ref21] Van Vliet T (2001). Homesickness: Antecedents, consequences, and mediation processes.

[ref22] Yeh C, Inose M (2003). International students reported English fluency, social support satisfaction, and social connectedness as predictors of acculturative stress. Counseling Psychology.

